# Health and Well-Being of Cisgender, Transgender and Non-Binary Young People

**DOI:** 10.3390/ijerph15102133

**Published:** 2018-09-28

**Authors:** Marta Evelia Aparicio-García, Eva María Díaz-Ramiro, Susana Rubio-Valdehita, María Inmaculada López-Núñez, Isidro García-Nieto

**Affiliations:** 1 Department of Social, Work and Differential Psychology, Universidad Complutense de Madrid, 28223 Madrid, Spain; ediazram@psi.ucm.es (E.M.D.-R.); srubiova@psi.ucm.es (S.R.-V.); mariai04@pdi.ucm.es (M.I.L.-N.); 2 Servicio LGTBI de la Comunidad de Madrid, 28031 Madrid, Spain; isidrogarcianieto@gmail.com

**Keywords:** transgender, non-binary gender identity, adolescence, health, well-being, gender nonconforming

## Abstract

Young transgender and non-binary individuals (TNBI) are exposed to situations of discrimination and have a greater risk of violence. The purpose of this study is to analyze which protective, violence and health and well-being factors have more influence on TNBI compared to cisgender people. The sample comprised 856 youth between 14 and 25 years old. A survey including questions about sociodemographic information and protective, violence and health and well-being factors was designed ad hoc for this study. The results show the non-binary group received the least support from family and friends, higher risk of suffering cyberbullying, and many feel isolated and unhappy. TNBI have suffered more verbal attacks both inside and outside their school and physical attacks at school than cisgender young. These results are important because they may contribute to the promotion of public policies and clinical interventions that favor the integration of TNBI in our society.

## 1. Introduction

The term *transgender* is used inclusively to describe individuals who have “gender identities, expressions, or behaviors not traditionally associated with their birth sex” [1]. *Non-binary* term refers to a person whose gender identity falls outside of the gender binary (i.e., identifies with neither or both genders) [2].

The legal situation of transgender people in Spain has improved over the last few decades, but it is still complex today. The Law 3/2007 on the rectification of the mention of the sex of the people (Law 3/2007, of 15 March, Regulatory Registration Rectification of the Mention Relative to the Sex of the People, BOE n°65) established the public regulation of Legal Gender Recognition (LGR). This law represented an important step in the legal framework in Spain, since it established the necessary requirements for civil registration of the LGR based on the principle of the right to privacy and guaranteed to trans people the possibility of exercising all the rights inherent in their new legal condition, such as a change of name and gender without the mandatory requirement of genital surgery nor forced sterilization (previously required). However, a psychiatric diagnosis is still mandatory and the law forces trans people to certify two years of medical treatment, although not all trans people need it or want it, which can cause the development of pathologies in these people.

Even with the existence of some legislation recognizing transgender rights and advocating for more social tolerance, there is also abundant evidence that Spanish society still exhibits prejudice and discrimination. For example, in a study published by the Federation of Lesbians, Gays, Transsexuals, and Bisexuals [3], it was found that 30% of young Lesbians, Gays, Bisexuals and Transsexuals people (LGBT) had suffered bullying due to their sexual orientation frequently, and among them, 43% had thought about suicide, 35% had planned it, and 17% had attempted it on various occasions. In addition, between 11% and 18% of young Spanish people consider it right to treat people with contempt because of their gender identity [3].

The Minority Stress Model [4] postulates that sexual minorities experience increased mental health problems because of stress processes unique to their status, namely discrimination, expectations of rejection, concealment, and internalized homophobia. Integrating the Minority Stress Model and the Psychological Mediation Framework [5], The Health Equity Promotion Model [6] adds a life course development perspective within a health equity framework to highlight how social positions, individual and structural, environmental contexts and health-promoting, and adverse pathways to influence the continuum of health outcomes in LGBT communities. Based in these models our main objective is analyze the influence of violence, protective, health, and well-being factors on young transgender and non-binary individuals (TNBI) compared to cisgender individuals.

Adolescents and transgender youth populations are especially exposed to situations with risk of violence [7]. Despite this, few studies have focused specifically on this population. In most studies that combine LGBT adolescents with transgender youth samples, differences between social support and risk of violence between both groups have been found. Results indicate that transgender adolescents show an increased risk of mistreatment, self-harm, depression [8], and suicide [9].

Despite, there are some protective factors that can help to TNBI. For example, the social support of parents operates as a protector against depressive symptoms; the perceived burden of being transgender is associated with greater life satisfaction [10]. Social support has been considered a moderating variable of the negative effects of discrimination on health and well-being. Social support refers to the set of contributions of an emotional, material, informational or company type that people perceive or receive from different members of their social network [11]. Feelings of being appreciated by other people and belonging to a social network can influence well-being and health, either directly or through other psychological processes. Social support is especially important in a moment of stress generated by situations of discrimination, influencing in a positive way well-being and the ability toe cope with stressful situations [12].

Studies with adolescents find a relationship between perceived social support and adjustment [13,14], as well as between social support and informed health problems, although in this case relationships are usually low [15,16]. Specifically, in transgender people, studies indicate that social support and acceptance are positively related to mental health [17,18]. Among the transgender population, family rejection has been related to a number of risk factors, such as homelessness, sex work, and suicidality [17,19].

Social support for the transgender collective is a particularly relevant factor to speak out about political concerns and use social networks [20]. Studies indicate that transgender individuals show lower scores in social support than cisgender [21]. Transgender perceived less social support from their family members than cisgender siblings [22]. Despite these results that indicate that social support is a variable that reduces distress, in transgender people it is not clear what kind of social support (e.g., family, friends, romantic) is related to decreases in suffering [23]. In a recent meta-analysis of the relationships between multiple forms of perceived discrimination in both mental and physical health outcomes of discriminated groups, results showed that protective effects depend on several variables, such as the level of perceived discrimination, type of well-being evaluated, and the kind of social support [24].

TNBI are particularly vulnerable to mental health concerns and psychological distress [25,26,27,28], elevated risk of depression [29,30], high rates of prevalence in anxiety [31], and less self-esteem [32]. The experiences of discrimination and transphobia have been closely linked to attempted suicide and non-suicidal self-harm [33,34]. Therefore, the rates of victimization and intimidation experienced by these people increase the risk of depression, anxiety and other risk factors for suicidal ideation [35].

Some studies show that transgender people are less likely to be engaged in regular physical activity and reported significantly poorer physical health than cisgender people [6]. Health behaviors that are likely to affect transgender health include higher rates of smoking [36], alcohol [31,37], and drugs [38].

Research in transgender adults analyzing relationships between social support and physical and psychological health indicate that transgender people show lower levels of social support than cisgender adults, although their social network is greater. Regarding transgender groups, social support is associated with better physical health, less likelihood of discomfort, and lower scores of depressive symptoms and stress [6].

Another study analyzed a sample of New Zealanders transgender and cisgender adolescents in relation to protective factors, health, and psychological well-being [39], most of the transgender adolescents surveyed report that they had at least one parent who cared for them, that they felt safe in their school and neighborhood, and that they were not suicidal and did not have significant depressive symptoms.

TNBI are exposed to situations of discrimination and have a greater risk of violence, despite this, there are very few studies that analyze the health and well-being of TNBI. The purpose of the current study is to analyze which violence, protective and health and well-being factors have more influence on TNBI compared to cisgender people. Four hypotheses are evaluated: (1) TNBI have more risk of violence and more employment discrimination than cisgender people; (2) TNBI receive less support from family and friends and participate less in social activities than cisgender people; (3) TNBI have worse health indicators than cisgender people.

## 2. Method

### 2.1. Participants

Participation in the study was offered to 856 young people between 14 and 25 years old and 782 responded to the survey (91.35% response rate). The mean age of participants was 20.36 years (SD = 3.12). Of the total sample 532 were identified as cisgender (68%), 180 as transgender (23%) and 70 as non-binaries (9%). 73% of the sample (*n* = 574) indicated to be atheist, 15% (*n* = 120) Catholic, 6% (*n* = 46) agnostic and 5% (*n* = 42) reported having another religion (see Table 1).

### 2.2. Procedure

Participants were recruited through websites, Twitter, and different associations (Daniela Foundation, FELGTB and COGAM) in Spain. First, we contacted the organization and explained the goals of the research and the method. The survey was included in an online survey (Google Docs, Android version 1.6.292). The organization then provided the link through its own website, Twitter or Facebook, so that any person who accessed the site could complete the survey, regardless of if they were members of these associations. Participants read a brief instruction describing the research and agreed to participate before answering the survey. Participation in the research was anonymous and voluntary and we asked about “consent to participate”. All procedures performed in this study were in accordance with the ethical standards of the institutional and/or national research committee and with the 1964 Helsinki declaration and its later amendments or comparable ethical standards.

### 2.3. Instrument 

A survey including questions about sociodemographic information and protective, violence and health and well-being factors was designed ad hoc for this study.

The first part of the survey included questions about gender, age, residence, and religion. Whether a participant was transgender was measured by the question, “Do you consider yourself?” (with response options of “male”, “female”, “transman”, “transwoman” or “non-binary”). Participants were categorized into one of three gender groups: cisgender (male and female), transgender (transman and transwoman) or non-binary. The type of residence area of the participant where people living were measured with the question “Would you describe the area in which you live?” (with response options of “rural” or “urban”). The religion with which they identified was measured by the question “What is your religion?” (with the response options “Catholic”, “Jewish”, “Muslim”, “Evangelist”, “Other”, “Atheist”, “Agnostic” or “I prefer not to answer”).

To assess social integration and participation, harassment and /or exclusion, social support, self-esteem and general health, a survey of 24 dichotomous response questions (SI/NO) was designed with three indicators: *Protective factors* with 9 questions (e.g., “*I have support from my family*”), *Violence or Personal safety* with 7 questions (e.g., “*I have been verbally harassed at school*”) and *Health and Well-being* with 8 questions (e.g., “*Once I have thought about suicide*”). To obtain an additional mental health indicator the General Health Questionnaire (GHQ-12) questionnaire was included at the end of the survey. The 12-Item GHQ-12 [40] has demonstrated adequate reliability and validity to detect mental health problems and assess psychological distress in the Spanish population [41].

### 2.4. Data Analysis

Associations between gender and protective, violence and health and well-being factors were analyzed by logistic regression using SPSS 22.0 version (IBM, Madrid, Spain). Participant’s age, residence and religion were included as covariates in the logistic regression models to control a possible moderating effect. Cisgender was included in all analysis as the reference group.

## 3. Results

TNBI were at increased risk of violence and employment discrimination (first hypothesis). For example, between approximately 40 and 50% of TNBI have suffered verbal attacks both inside and outside their school and physical attacks at school are significantly more frequent for these groups than for cisgender people. Non-binary groups shows higher risk of suffering cyberbullying (Table 2).

All protective factor considered in the study result significant (second hypothesis). Non-binary group received the least support from family and friends and was the one that least participated in the different activities that take place in their social environment (Table 3).

In terms of health indicators (third hypothesis), no differences were found between the groups in drug use or in smoking, and TNBI consume less alcohol than cisgender. However, in comparison with the cisgender group, people in the other groups had increased psychological health and well-being needs. TNBI show a higher percentage of people who feel isolated and unhappy, have more psychological health problems according to the GHQ-12 questionnaire, and seven in ten have thought about suicide (Table 4).

## 4. Discussion

In this research, we analyzed gender groups and associations with protective factors, violence or personal safety variables, and health and well-being indicators in three different groups (cisgender, transgender and non-binary) of young people in Spain. 

Regarding the factors of violence or personal safety (first hypothesis), TNBI were at an increased risk of violence [30,42] and only the non-binary group shows higher risk of suffering cyberbullying, which is consistent with previous studies on the LGBT population [43]. Like previous research [44], TNBI have felt discriminated against when looking for a job.

TNBI receive less support from family and friends and participate less in social activities than cisgender people. In terms of protective factors (second hypothesis), our group of non-binary people received the least support from family and friends. This is in line with previous research showing that gender nonconforming young people feel less support than other sexual minorities [39,45] and report experiences with family rejection [19,46,47]. Non-binary people were the ones that least participated in the different activities in their social environment. This result was found previously in Australian research: non-binary people participated less than cisgender people in the LGBT community [48].

In terms of health and well-being indicators (third hypothesis), TNBI consume less alcohol than cisgender. That is a positive result of our research that it is different than that obtained in other countries. Studies across North America suggest that drug (including nicotine) and alcohol use is common among transgender individuals [29,31,37], but this research has not been done with young people. A recent Australian study show 11% of the transgender young people felt they had addictions (23% took drugs, 26% smoke cigarettes, and 48% drank alcohol) [49]. Also, our TNBI show more psychological health problems and have ever thought about suicide more frequently than cisgender people. Estimates of suicidal ideation and suicide attempts in transgender people vary widely; in fact, since 2000 there have been rates ranging from 11% to 43% in suicidal attempts and from 7% to 89% for suicidal ideation [50]. From some studies it arises that the most vulnerable groups to attempt suicide where young people between 16 and 24 years (19%), especially those who experienced transphobic, physical or sexual violence (28.8%), and those who have only just begun a medical transition (26.6%) [33]. A long history of suppression of transgender feelings may have resulted in isolation, loneliness, and feelings of hopelessness; the fear of disclosing this secret to partners, family, friends, and co-workers-risking rejection and employment discrimination-can provoke a great deal of anxiety [25]. Our TNBI show a higher percentage of people who feel isolated and unhappy.

## 5. Limitations and Future Research

Our study has some limitations that should be overcome in future studies. First, despite the number of participants, the sample should be expanded to improve the generalizability of the results. We must not forget that the information was obtained through the collaboration of LGTB association (although not alone) and this could bias the results, leaving out of the study people that have no Internet access or who are not involved in the associative sector. Second, as the survey contained many sensitive topics, young people could choose not to answer a question. This could be the reason why there was a small percentage of missing data. Third, in our study we asked about the variables of the study through a survey, but not with a complete questionnaire that measured each of the variables, so it would be advisable in future investigations to use a broader questionnaire to deepen the answers of participants, because there could be a certain acquiescence in yes/no responses. Finally, due to the fact our study is quantitative, some questions have not been resolved; for example, why non-binary people are the least involved in social activities. It would be interesting to analyze in future studies the reasons why they do not participate as much as the other groups through a qualitative study.

## 6. Conclusions

The present study is one of the first performed in Spain to analyze the health and well-being of transgender, and especially non-binary young people. Our results are consistent with previous studies when pointing out the positive effects of social support in sexual minorities [51]. The situation of transgender people in the world varies according to the policies established by governments [52] and our results justify the need to promote public policies that favor the integration of transgender people in our society. It is therefore necessary that, as soon as possible, a new law should be passed (modifying the current LGR pathologizing state law) establishing a fair and equitable process for all transgender people in Spain. A single framework that regulates rights and obligations for transgender people is required. The rupture of binary gender models is a reality in our society, as shown by different studies that find that half the transgender student population were non-binary [49]. Finally, greater acceptance among the general population and understanding of the experiences of transgender communities may help to reduce the occurrence of transphobic events, as indicated by previous studies [53].

## Figures and Tables

**Table 1:** Demographic characteristics of students by gender group.

Demographic Characteristics	Gender Subgroup		
	Transgender, *n* (%)	Non-Binary, *n* (%)	Cisgender, *n* (%)
Sexual Orientation			
Heterosexual	83 (47.2)	2 (2.9)	181 (34.2)
Gay	5 (2.8)	3 (4.4)	91 (17.2)
Lesbian	4 (2.3)	10 (14.7)	93 (17.6)
Bisexual	21 (11.9)	10 (14.7)	99 (18.7)
Pansexual	55 (31.3)	32 (47.1)	46 (8.7)
Other	4 (2.3)	10 (14.7)	14 (2.6)
Missing	4 (2.3)	1 (1.3)	5 (0.9)
Religion			
Catholic	32 (17.8)	2 (2.9)	86 (16.2)
Atheist or agnostic	117 (65)	55 (78.6)	402 (75.6)
Other	13 (7.2)	10 (14.3)	23 (4.3)
Missing	18 (10)	3 (4.3)	21 (3.9)
Area			
Rural	35 (5.7)	4 (5.7)	60 (11.3)
Urban	143 (79.4)	65 (92.9)	470 (88.3)

**Table 2:** Associations between gender group and violence or personal safety.

Violence or Personal Safety	*n* (%)	Odds Ratio (95% Confidence Interval)	*p*
Excluded by your peer group at some time			0.167
Cisgender (*n* = 526)	128 (24.3%)	1.00	
Transgender (*n* = 120)	22 (18.3%)	0.63 (0.37–1.08)	
Non-binary (*n* = 41)	8 (19.5%)	0.67 (0.28–1.58)	
Verbal attacks at school			0.000
Cisgender (*n* = 524)	132 (25.2%)	1.0	
Transgender (*n* = 165)	71 (43.0%)	1.74 (1.16–2.61)	
Non-binary (*n* = 68)	29 (42.6%)	1.99 (1.14–3.45)	
Verbal attacks out of school			0.000
Cisgender (*n* = 527)	155 (29.4%)	1.0	
Transgender (*n* = 173)	76 (43.9%)	1.87 (1.28–2.76)	
Non-binary (*n* = 68)	38 (55.9%)	3.17 (1.84–5.45)	
Physical attacks at school			0.000
Cisgender (*n* = 527)	43 (8.2%)	1.0	
Transgender (*n* = 170)	38 (22.4%)	2.72 (1.59–4.63)	
Non-binary (*n* = 66)	9 (13.6%)	1.94 (0.88–4.29)	
Physical attacks out of school			0.018
Cisgender (*n* = 340)	82 (24.1%)	1.0	
Transgender (*n* = 173)	27 (15.6%)	0.60 (0.37–0.99)	
Non-binary (*n* = 65)	8 (12.3%)	0.46 (0.21–1.02)	
Cyberbullying			0.000
Cisgender (*n* = 479)	144 (30.1%)	1.0	
Transgender (*n* = 177)	37 (20.9%)	0.46 (0.29–0.73)	
Non-binary (*n* = 70)	29 (41.4%)	1.49 (0.87–2.57)	
Discrimination when looking for a job			0.000
Cisgender (*n* = 462)	95 (20.6%)	1.0	
Transgender (*n* = 133)	71 (53.4%)	6.81 (4.18–11.09)	
Non-binary (*n* = 49)	27 (55.1%)	6.56 (3.31–13.00)	

**Table 3:** Associations between gender group and protective factors.

Protective Factors	*n* (%)	Odds Ratio (95% Confidence Interval)	*p*
Involvement in extracurricular activities in school			0.018
Cisgender (*n* = 517)	181 (35.0%)	1.0	
Transgender (*n* = 166)	46 (27.7%)	0.47 (0.27–0.80)	
Non-binary (*n* = 67)	16 (23.9%)	0.52 (0.23–1.19)	
Practice of a sport in school or outside it			0.000
Cisgender (*n* = 532)	248 (46.6%)	1.0	
Transgender (*n* = 180)	89 (49.4%)	0.12 (0.03–0.52)	
Non-binary (*n* = 70)	19 (27.1%)	0.07 (0.01–0.47)	
Go out with friends			0.000
Cisgender (*n* = 532)	456 (85.7%)	1.0	
Transgender (*n* = 180)	133 (73.9%)	0.19 (0.02–2.30)	
Non-binary (*n* = 70)	44 (62.9%)	0.09 (0.01–1.50)	
Take part in LGBT associations			0.000
Cisgender (*n* = 344)	84 (24.4%)	1.0	
Transgender (*n* = 175)	75 (42.9%)	1.58 (0.83–3.00)	
Non-binary (*n* = 66)	19 (28.8%)	0.57 (0.26–1.28)	
Take part in online LGTB groups			0.000
Cisgender (*n* = 341)	102 (29.9%)	1.0	
Transgender (*n* = 174)	78 (44.8%)	0.32 (0.08–1.30)	
Non-binary (*n* = 66)	43 (65.2%)	0.55 (0.10–3.04)	
Family support			0.000
Cisgender (*n* = 317)	186 (58.7%)	1.0	
Transgender (*n* = 168)	86 (51.2%)	0.84 (0.56–1.27)	
Non-binary (*n* = 53)	14 (26.4%)	0.26 (0.13–0.51)	
Adult support outside the family			0.015
Cisgender (*n* = 292)	169 (57.9%)	1.0	
Transgender (*n* = 168)	98 (58.3%)	1.23 (0.80–1.88)	
Non-binary (*n* = 59)	25 (42.4%)	0.48 (0.26–0.88)	
Friends support			0.000
Cisgender (*n* = 325)	312 (96.0%)	1.0	
Transgender (*n* = 174)	153 (87.9%)	0.31 (0.14–0.70)	
Non-binary (*n* = 64)	54 (84.4%)	0.22 (0.08–0.57)	
Have a remunerated job			0.000
Cisgender (*n* = 532)	126 (23.7%)	1.0	
Transgender (*n* = 180)	23 (12.8%)	0.87 (0.50–1.51)	
Non-binary (*n* = 70)	6 (8.6%)	0.31 (0.11–0.90)	

**Table 4:** Associations between gender group and health or well-being indicators.

Health or Well-Being Indicators	*n* (%)	Odds Ratio (95% Confidence Interval)	*p*
Feeling isolated			0.000
Cisgender (*n* = 355)	103 (29.0%)	1.0	
Transgender (*n* = 176)	71 (40.3%)	1.35 (0.90–2.04)	
Non-binary (*n* = 70)	38 (54.3%)	2.61 (1.50–4.52)	
Ever think about suicide			0.000
Cisgender (*n* = 520)	211 (40.6%)	1.0	
Transgender (*n* = 172)	121 (70.3%)	2.82 (1.89–4.20)	
Non-binary (*n* = 68)	53 (77.9%)	4.43 (2.40–8.18)	
Tried drugs and alcohol			0.365
Cisgender (*n* = 521)	308 (59.1%)	1.0	
Transgender (*n* = 176)	85 (48.3%)	0.81 (0.55–1.18)	
Non-binary (*n* = 68)	36 (52.9%)	0.74 (0.43–1.27)	
Psychological health problems			0.000
Cisgender (*n* = 529)	203 (38.4%)	1.00	
Transgender (*n* = 176)	92 (52.3%)	1.56 (1.07–2.26)	
Non-binary (*n* = 70)	39 (55.7%)	1.76 (1.04–2.99)	
Smoke			0.655
Cisgender (*n* = 531)	133 (25.0%)	1.0	
Transgender (*n* = 180)	45 (25.0%)	1.04 (0.68–1.59)	
Non-binary (*n* = 70)	15 (21.4%)	0.76 (0.40–1.45)	
Drank alcohol			0.009
Cisgender (*n* = 532)	342 (64.3%)	1.0	
Transgender (*n* = 179)	76 (42.5%)	0.55 (0.38–0.81)	
Non-binary (*n* = 70)	40 (57.1%)	0.75 (0.44–1.29)	
Drug use			0.992
Cisgender (*n* = 531)	146 (27.5%)	1.0	
Transgender (*n* = 179)	43 (24.0%)	1.02 (0.67–1.55)	
Non-binary (*n* = 70)	20 (28.6%)	1.03 (0.57–1.85)	
Happy or very happy			0.000
Cisgender (*n* =492)	411 (83.5%)	1.0	
Transgender (*n* = 165)	109 (66.1%)	0.49 (0.32–0.76)	
Non-binary (*n* = 57)	35 (61.4%)	0.38 (0.21–0.70)	
